# Cholesteryl ester levels are elevated in the caudate and putamen of Huntington’s disease patients

**DOI:** 10.1038/s41598-020-76973-8

**Published:** 2020-11-20

**Authors:** Gabrielle R. Phillips, Sarah E. Hancock, Simon H. J. Brown, Andrew M. Jenner, Fabian Kreilaus, Kelly A. Newell, Todd W. Mitchell

**Affiliations:** 1 Illawarra Health and Medical Research Institute, Wollongong, NSW 2522 Australia; 2 School of Medicine, University of Wollongong, Wollongong, NSW 2522 Australia; 3 Molecular Horizons, University of Wollongong, Wollongong, NSW 2522 Australia; 4 School of Medical Sciences, University of New South Wales, Sydney, NSW 2052 Australia; 5 School of Chemistry and Molecular Biosciences, University of Wollongong, Wollongong, NSW 2522 Australia; 6 Bioanalytical Mass Spectrometry Facility, Mark Wainwright Analytical Centre, University of New South Wales, Sydney, NSW 2052 Australia; 7 School of Medicine, Western Sydney University, Sydney, NSW 2560 Australia

**Keywords:** Lipids, Metabolomics, Neurochemistry, Neurological disorders, Diseases of the nervous system, Molecular neuroscience

## Abstract

Huntington’s disease (HD) is an autosomal dominant neurodegenerative illness caused by a mutation in the huntingtin gene (HTT) and subsequent protein (mhtt), to which the brain shows a region-specific vulnerability. Disturbances in neural cholesterol metabolism are established in HD human, murine and cell studies; however, cholesteryl esters (CE), which store and transport cholesterol in the brain, have not been investigated in human studies. This study aimed to identify region-specific alterations in the concentrations of CE in HD. The Victorian Brain Bank provided post-mortem tissue from 13 HD subjects and 13 age and sex-matched controls. Lipids were extracted from the caudate, putamen and cerebellum, and CE were quantified using targeted mass spectrometry. ACAT 1 protein expression was measured by western blot. CE concentrations were elevated in HD caudate and putamen compared to controls, with the elevation more pronounced in the caudate. No differences in the expression of ACAT1 were identified in the striatum. No remarkable differences in CE were detected in HD cerebellum. The striatal region-specific differences in CE profiles indicate functional subareas of lipid disturbance in HD. The increased CE concentration may have been induced as a compensatory mechanism to reduce cholesterol accumulation.

## Introduction

Huntington’s disease (HD) is an autosomal dominant, neurodegenerative disorder, affecting approximately 10 people per 100,000 in Western countries^1,2^. The disease is caused by a mutation in the Huntingtin gene (HTT), which results in a polyglutamine expansion (CAG repeats) in the huntingtin protein (htt). Disease manifestation is dependent on the number of CAG repeats^3^. Less than 35 repeats do not result in disease, 36–39 may or may not result in HD, while 40–59 leads to adult-onset and more than 60, juvenile-onset^2,4^. In adult-onset HD, symptoms usually appear around the age of 40 and progress consistently until death approximately 10–25 years later^1^. These symptoms include cognitive (dementia and difficulty organizing thoughts), psychiatric (anxiety and depression) and motor (chorea and dystonia)^1,2^. The most prominent site of neurodegeneration in the HD brain is the striatum, an area responsible for voluntary movement with evidence emerging for roles in memory, learning and social cognition^2,5–8^. The striatum contains three smaller subregions; the caudate, putamen and nucleus accumbens^8^. The medium spiny neurons of the striatum have a selective vulnerability to the mutant huntingtin protein (mhtt) that does not affect other neuronal subpopulations as severely, irrespective of their higher mhtt concentrations^3^. The striatum suffers mass decreases of up to 50%, a known pathological hallmark of HD^7^. Although the cause of HD has been identified (mhtt), the mechanism of pathology and specific interference with the striatum is unknown, resulting in no effective treatment for HD^2^. For this reason, research over the last twenty years has focused on determining if the molecular environment of certain brain regions is responsible for their vulnerability to mhtt.

**Figure 1:**
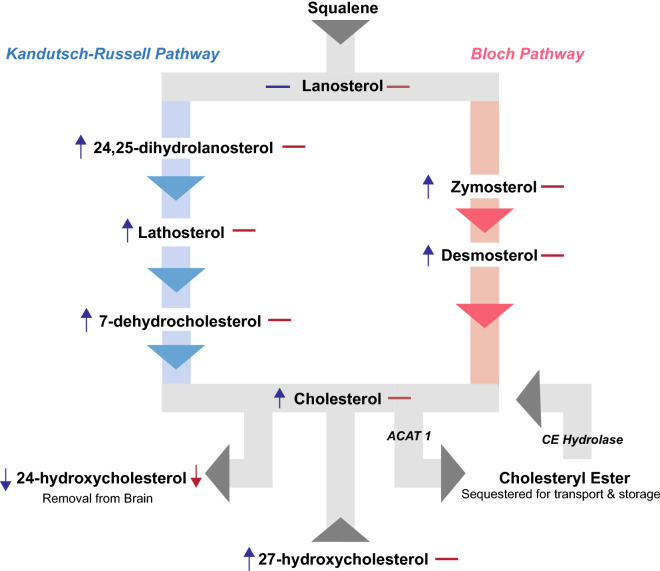
Simplified cholesterol metabolic pathway. Cholesterol can be synthesised via either the Kandutsch–Russell Pathway (on left) or the Bloch Pathway (on right). Symbols next to each metabolite indicate whether it has been identified as either increased (up arrow), decreased (down arrow) or unchanged (dash) in the putamen (blue) and caudate (red) human post-mortem tissue (Kreilaus et al. 2016). *ACAT 1* acyl-coenzyme cholesterol acyltransferase, *CE* cholesteryl ester.

Lipids are a significant part of the neural molecular environment, constituting approximately 50% of the dry weight of the brain^9^. Lipids are essential for proper brain function; required for axon growth and potentiation, membrane trafficking, myelin formation, synaptogenesis and are a critical component of cell membranes^10–13^. Depending on the cellular location in the brain, concentration and synthesis of lipids such as cholesterol, differ depending on specific structural and functional requirements^14^. Cholesterol is an abundant neural lipid, with disturbances in related metabolic pathways identified in HD transgenic mice, striatal cells, and human post mortem tissue^7,12,15–17^. It is primarily synthesised by local de novo synthesis in the brain and cannot readily cross the blood–brain barrier (BBB). The conversion of cholesterol to 24-hydroxycholesterol (24-OHC) catalysed by the neuronal enzyme CYP46A1, therefore provides a crucial pathway for its removal from the brain and maintenance of critical homeostasis^13,15,18^. Cholesterol is most abundant in the striatum, likely due to the striatum’s high number of interconnections, which may underlie the specific vulnerability of the striatum to cholesterol dysfunction in HD^10,19^. Reductions to cholesterol precursors, particularly lanosterol and lathosterol have been identified in the striatum and cortex of several HD murine models including R6/1, R6/2 transgenic, YAC128, and Q175 knock-in models, and in the putamen of human HD post-mortem tissue^7,10,15,17,19^. Of these murine studies, Kreilaus et al. (2015) and Shankaran et al. (2017) both noted more substantial lipid disturbances to the striatum but only at the advanced stages of the disease, albeit using different murine models (R6/1 and Q175, respectively)^15,19^. Figure 1 shows a simplified cholesterol synthesis pathway with identified reductions/increases in metabolites as identified in HD human post-mortem tissue. Cholesterol accumulation has been identified in human HD putamen, in mouse models, and cultured HD striatal neurons^7,15,20^.

There is evidence of a reduced ability to remove cholesterol from the brain via 24-OHC in HD, with 24-OHC reductions identified in human caudate, putamen and plasma, with plasma concentration of 24-OHC directly correlating with caudate degeneration^7,18,21^. Previous analysis of the caudate and putamen tissue used in this study identified reductions in 24-OHC in HD, with only the putamen showing increased cholesterol indicating that these subregions may have unique disturbances^7^. If cholesterol removal from the brain is impacted, cholesterol may be redirected into other downstream metabolites to prevent accumulation. Cholesteryl esters (CE) are a downstream metabolite of cholesterol, yet to be studied in the HD brain, and their examination would provide richer insight into cholesterol metabolism in HD. In the human brain, CE are sequestered as lipid droplets, primarily used for the transport and storage of cholesterol^13,16^. In the brain they are formed via the esterification of a fatty acid to cholesterol by an isoform of acyl coenzyme-A cholesterol acyltransferase (ACAT 1), a process proposed to occur allosterically in response to surplus cholesterol within the cell^22^ (Fig. 1). CE are increased not only in HD striatal cell models, but also in several neurological disorders such as multiple sclerosis, Alzheimer’s disease, brain injury and stroke^23–26^. Although the fatty acyl chains of multiple lipid classes determine specific biological functions^27–29^, no current evidence exists to support any specific functions of individual CE species. This study aimed to determine if CE are altered in HD striatum and to investigate if the striatal subregions (caudate and putamen) have unique disturbances in CE.

## Results

### Regional differences in total CE between control and HD tissue

**Figure 2:**
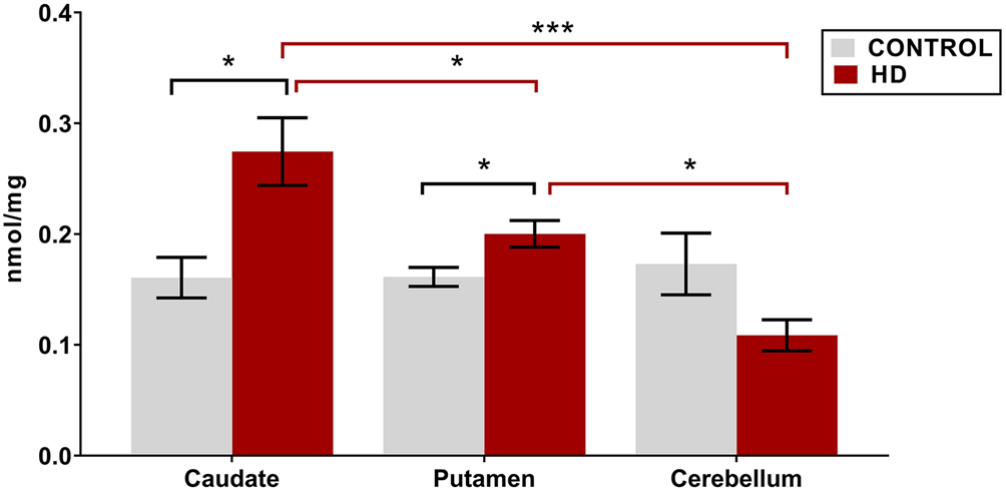
Total CE concentration in HD vs control in the caudate (n = 11), putamen (HD n = 12, control n = 11) and cerebellum (HD n = 9, control n = 12). Lipid concentrations are expressed as means ± standard error of the mean (nmol/mg brain tissue). Data was assessed for normality using a D’Agostino Pearson Omnibus test. Data was analysed using a two-way ANOVA with a Tukey’s test for multiple comparisons between regions. *p < 0.05, ***p < 0.0001. *CE* cholesteryl ester, *HD* Huntington’s disease.

Analysis of total brain CE between the caudate, putamen and cerebellum demonstrated interesting region-specific differences between control and HD tissues (Fig. 2). While no differences were observed in total CE between caudate, putamen and cerebellum within the control tissues, total CE levels were dysregulated between the three regions in the HD patients. For HD tissues, the caudate contained the highest amount of CE compared with both the putamen (+ 27%, p = 0.0360) and cerebellum (+ 60%, p = 0.0001). Within each region of the brain, both the caudate and putamen contained higher amounts of total CE than their corresponding control tissues (+ 27%, p = 0.0045, and + 10%, p = 0.0181, respectively), while no statistically significant differences were observed in total CE between HD and control in the cerebellum. Polyglutamine repeat numbers were not provided in the clinical information. Therefore, a correlation analysis was run to examine if total CE levels were correlated with age of death in HD subjects (Supplementary Table S1), as there is direct relationship between polyglutamine repeat number and age of death^30^. No correlations were observed. Additional correlation analyses were run to determine relationships between biometric data, disease status and total CE (Supplementary Tables S1, S2, and S3). No correlations were observed.

### Differences in molecular CE species between control and HD samples

**Figure 3:**
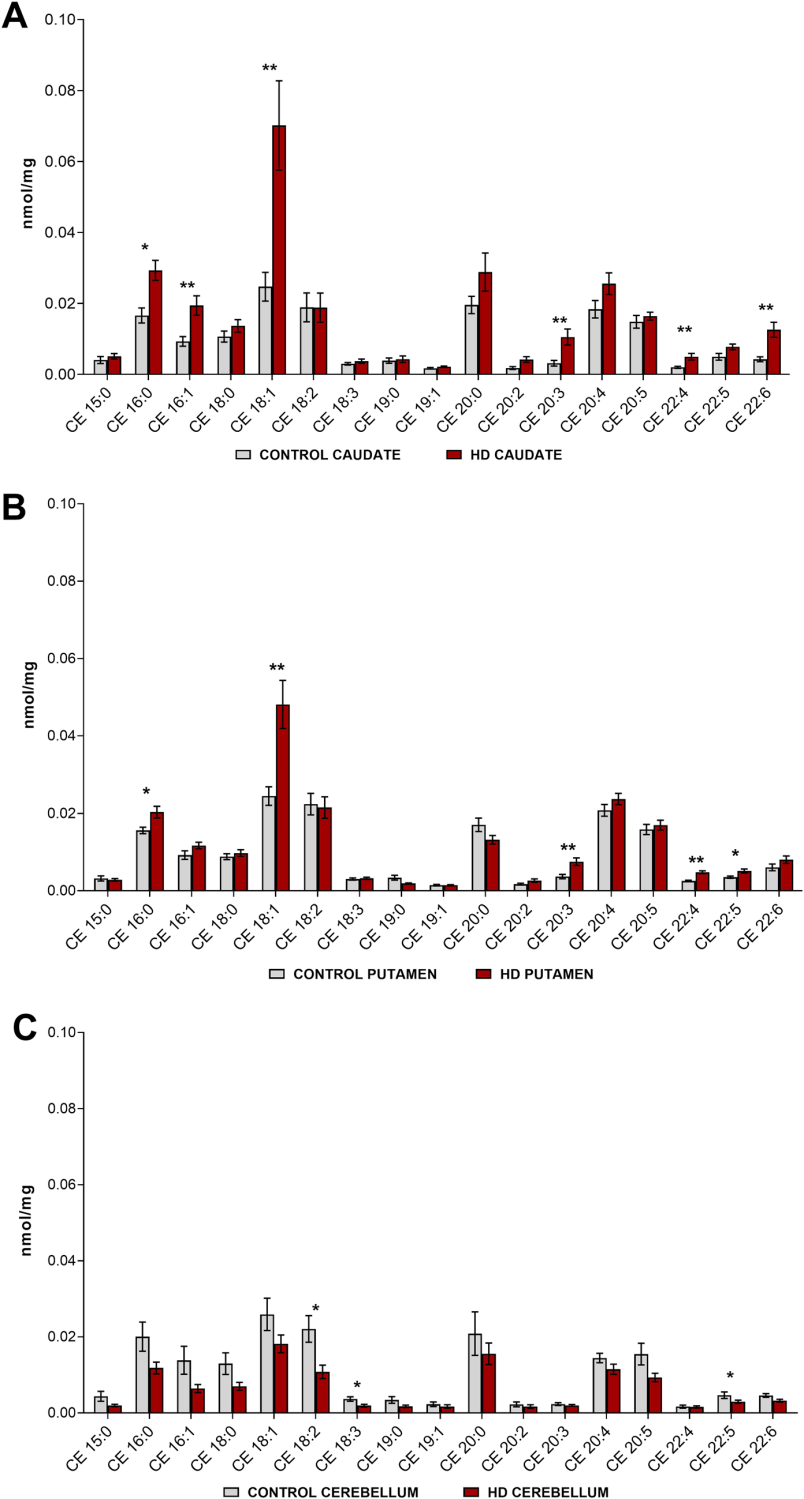
Concentrations of CE species in HD vs. control in (**a**) Caudate (HD and control n = 11). (**b**) Putamen (HD n = 13, control n = 11). (**c**) Cerebellum (HD n = 9, control n = 12). Nomenclature of CE is determined as the number of carbons in the fatty acyl chain and the number of double bonds (i.e. 16:1 has 16 carbons and 1 double bond). Lipid concentrations are expressed as means ± standard error of the mean (nmol/mg brain tissue). Data was assessed for normality using a D’Agostino Pearson Omnibus test. Data which was normally distributed was analysed using an unpaired t-test with Welch’s correction or a Mann–Whitney U test where data was not normally distributed, and the transformation was unsuccessful. *p < 0.05, **p < 0.01. *CE* cholesteryl ester, *HD* Huntington’s disease.

The nomenclature of CE species used in this paper identifies the number of carbons and double bonds of the fatty acyl chain i.e. 16:1 is a fatty acyl chain with 16 carbons and 1 double bond. Seventeen common species of CE were identified across all tissues (Fig. 3). The most dominant CE species in both the control and HD tissues across the three regions of the brain were typically 16:0, 18:1, 20:0, 20:4 and 20:5. Little variation in lipid species abundance was observed across the three control brain regions. Six species of CE were primarily responsible for the increase in total CE in HD caudate (Fig. 3a). Species affected included both long and very long-chain fatty acids, and all degrees of saturation (saturated, monounsaturated, and polyunsaturated). These species, with their percentage increase in concentration relative to controls were: CE 16:0 (+ 76%, p = 0.0018), CE 16:1 (+ 108%, p = 0.0034), CE 18:1 (+ 183%, p = 0.0026), CE 20:3 (+ 233%, p = 0.0006), CE 22:4 (+ 149%, p = 0.0024) and CE 22:6 (+ 195%, p = 0.0013).

CE species in the putamen also showed no distinct pattern of species affected (unsaturated, monounsaturated). Four of the same CE species that were increased in HD caudate, were also increased, but to a lesser extent in HD putamen: CE 16:0 (+ 30%, p = 0.0148), CE 18:1 (+ 96%, p = 0.0032), CE 20:3 (+ 105%, p = 0.0026) and CE 22:4 (+ 89%, p = 0.0001) (Fig. 3b). An increase in an additional CE species within the HD putamen, CE 22:5 (+ 46%, p = 0.0073) was also observed. Within the cerebellum, three species of polyunsaturated CE were found to be lower in abundance in HD: CE 18:2 (− 51%, p = 0.0205), CE 18:3 (− 47%, p = 0.0111) and CE 22:6 (− 30%, p = 0.0489) (Fig. 3c). High variability in cerebellar lipid profiles may be responsible for the failure to detect a difference in CE totals between HD and control samples for this region. The cerebellum showed a lower abundance of CE (− 13%, p = 0.1264) in HD compared to controls although this data was not found to be statistically significant. All CEs measured are listed in Supplementary Tables S4, S5 and S6.

### Region-specific comparison of alterations in CE to cholesterol and 24-OHC

**Figure 4:**
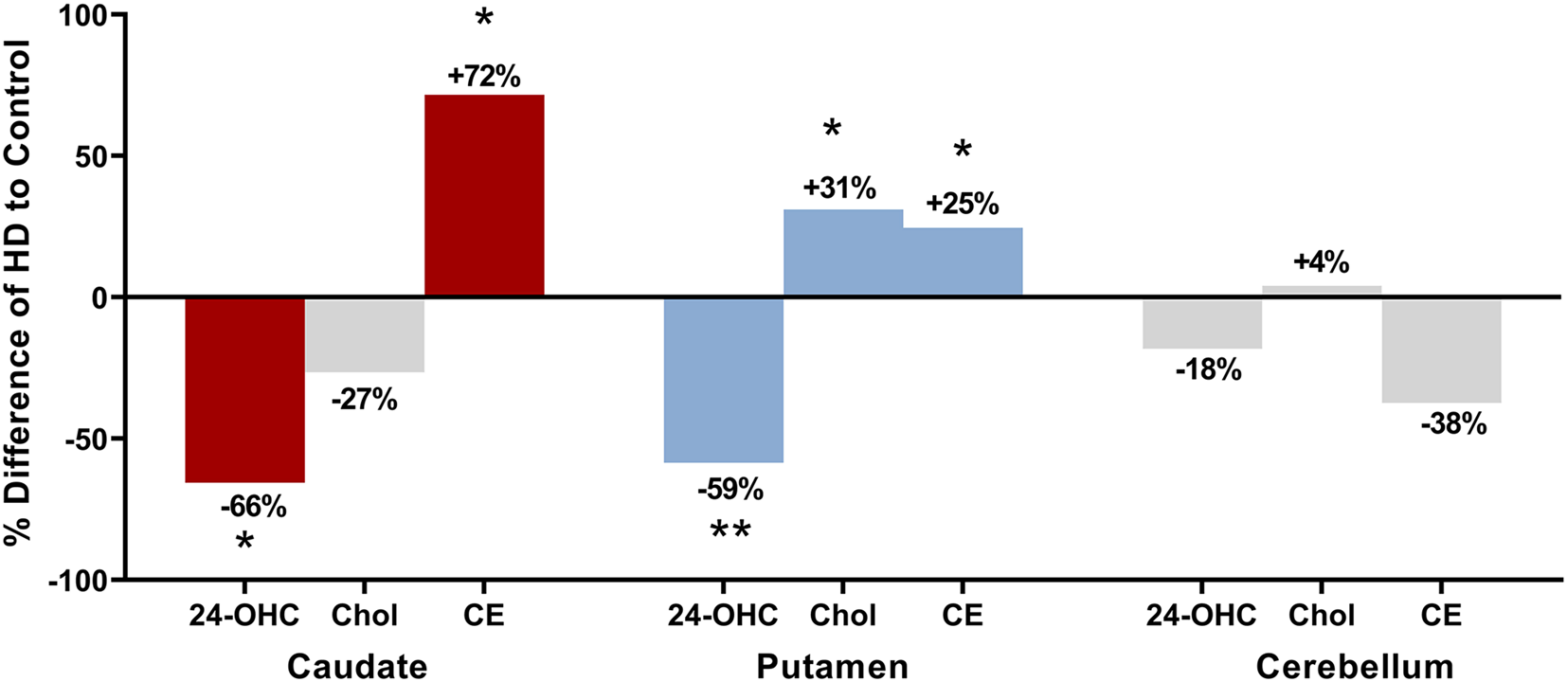
Changes to 24-hydroxycholesterol (24-OHC), cholesterol (Chol) and cholesteryl esters (CE) in brain regions. Percentage differences of HD values to Control values are shown with their respective percentage increases or decreases. Data from the previous analysis of the post-mortem tissue from this study was used for 24-OHC and cholesterol data (Kreilaus et al. 2016). Coloured bars are metabolite differences found to be statistically significant (24-OHC and cholesterol as determined by Kreilaus et al. 2016), whilst those in grey were not. The graph is showing that in HD caudate, the elevations in CE may have assisted in regulating cholesterol levels when removal via 24-OHC was reduced. HD putamen has a lower elevation in CE; however, it was found to have increases in cholesterol known to be detrimental to neural cells when homeostasis is disturbed. This graph is arguing for the use of CE as a compensatory pathway for cholesterol accumulation when removal is disturbed, and that HD caudate may be better at utilising this pathway than HD putamen. No significant disturbances were identified in HD cerebellum. *p < 0.05, **p < 0.01. *HD* Huntington’s disease.

Due to the alterations in cholesterol and 24-OHC found in the previous analysis of the tissue, differences in the total levels of CE in each region were compared. Figure 4 shows the percentage differences in 24-OHC, cholesterol and CE concentrations in HD relative to controls for all three regions. The analyses show that in HD caudate where cholesterol levels were not altered, 24-OHC, used for the removal of cholesterol was reduced (− 66%) along with a similar magnitude increase in CE (+ 72%, p = 0.0045). HD putamen did show an increase in cholesterol concentration (+ 31%), and a decrease in 24-OHC (− 59%) similar to HD caudate, although the increases in CE were not as substantial (+ 25%, p = 0.0181). HD cerebellum showed no differences in 24-OHC, cholesterol or total CE. Correlation analyses are available in Supplementary Table S7.

### ACAT1 expression was not different in HD striatum

**Figure 5:**
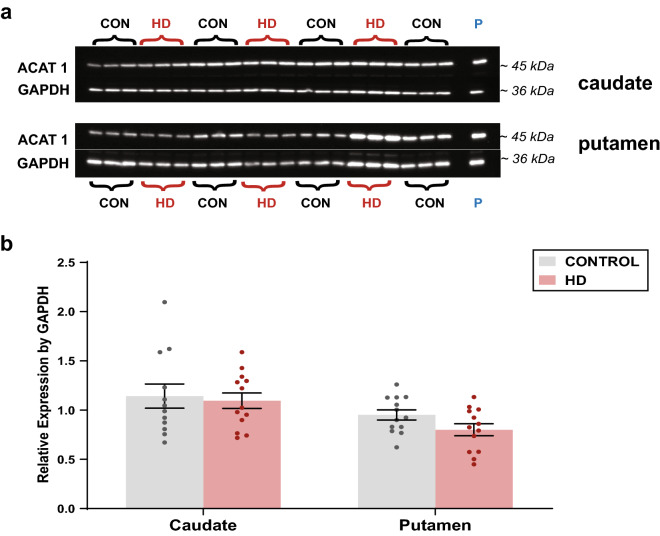
ACAT1 expression in the striatum (**a**) Western blot of caudate and putamen. Caudate and putamen samples were loaded onto separate membranes and each membrane was corrected for the ‘pooled’ sample and by GAPDH. Samples were loaded at 7.5 µg protein in triplicate. ACAT 1 expression detected as a single band at ~ 45 kDa. (**b**) Expression of ACAT1 in caudate and putamen relative to controls. A Two-Way ANOVA with a Tukey’s test was used to determine differences between groups (HD vs HD, HD vs CON). Data is expressed as the mean ± SEM relative to the expression of GAPDH. Individual subjects’ values are expressed as corresponding coloured symbols. Protein ladders are expressed in kDa. **p < 0.01. *ACAT1* acyl coenzyme-A cholesterol acyltransferase, *CON* control, *GAPDH* glyceraldehyde 3-phosphate dehydrogenase, *HD* Huntington’s disease.

Given the increased abundances of CE in HD caudate and putamen, we hypothesised that these abundances were due to increased expression of ACAT1. To test this, ACAT1 expression was determined in HD and control striatum. ACAT1 was detected as a single band at ~ 45 kDa (Fig. 5a). No difference in the expression of ACAT1 was detected between HD and control caudate or putamen (Fig. 5b).

Additionally, no differences were detected between HD caudate and putamen. Due to the absence of changes to total CE in HD cerebellum, ACAT1 expression was not measured in this region. Full western blot images are available for ACAT1 and GAPDH in the caudate (Supplementary Figs. S1 and S3) and in the putamen (Supplementary Figs. S2 and S4).

## Discussion

This study has identified an increased abundance of CE in HD striatum (caudate and putamen), the brain region most severely atrophied in HD^2,7^. Previous research in R6/1 and R6/2 HD murine models identified increases in key metabolites from both the Kandutsch-Russell and Bloch cholesterol synthesis pathways with little effect on steady-state levels of cholesterol, suggesting additional possible compensation mechanisms in the human brain. The current data suggest that the increased abundance of CE in HD striatum may represent the induction of a pathway that supports a capacity for cholesterol sequestration in the HD brain. The higher concentration of CE in HD caudate in the current study suggests an alternative compensatory pathway for sequestration of excess cholesterol through esterification if the 24-OHC pathway is disturbed. Although exhibiting similar decreases in the cholesterol removal product 24-OHC, the caudate and putamen show differences in CE and cholesterol. We suggest that HD caudate compensated cholesterol accumulation by sequestering it into cholesteryl esters. The putamen, which also had 24-OHC disturbed in a similar fashion to the caudate, had approximately half the increase of CE alongside cholesterol increases, indicating that this region may not have been able to compensate in the same way. ACAT1 activity is stimulated by a surplus of cholesterol in the cell, allowing for sequestration of cholesterol into esters which exist as lipid droplets in the cytoplasm^29,31^. We were unable to measure ACAT1 activity in this study therefore the possibility that ACAT1 activity is increased in HD cannot be ruled out. An alternative explanation may be an accumulation of CE over time. Synthesis and metabolism of CE is allosterically regulated^22,32^. Therefore when cholesterol excess subsides, CE are hydrolysed by cholesterol ester hydrolyase for conversion back to cholesterol^31^. However, this enzyme may have failed to either degrade excess CE in time or to override the continual formation of CE in response to high cholesterol within the cell. Recently, a study identified two additional configurations of ACAT1 as a dimer and a tetramer^32^. The study proposed the dimer as the active configuration, however, our protein analysis detected only the monomer. Although the accumulation of cholesterol in the cell is detrimental, the consequences of elevations in CE is still unknown.

Increases in CE have been found in the CNS of other neurodegenerative diseases such as multiple sclerosis (MS), amyotrophic lateral sclerosis (ALS), and brain injury, which may indicate CE accumulation as a general neuropathological consequence and not a feature of HD alone^23–25,33,34^. Specifically, in MS, CE are increased primarily in and surrounding white matter plaques. Analysis of human MS tissue identified no changes in the synthesis of CE, but instead a substantial reduction in the breakdown of CE, indicating that the high levels of CE were due to insufficient hydrolysis in MS^33^. Inhibition of ACAT1 activity can decrease the formation of amyloid-beta plaques in a mouse model of AD^35,36^. Therefore, CE is suspected to contribute to AD pathology. Cholesterol esterification in neurodegeneration may also prevent the synthesis of toxic cholesterol oxidation products, and therefore protect cells^23^. A study examining excitotoxic injuries in rat hippocampus suggested that raised CE concentrations are a direct result of increased cholesterol in response to neural injury^23^, which may be occurring in HD. Increases in CE have been observed in brain lesions that involved the breakdown of myelin^23^. Magnetic resonance imaging of HD patients has identified microstructural abnormalities in the white matter tracts which surround the striatum, indicating myelin breakdown^37^. These results support the hypothesis that CE may also be increased in HD striatum due to increased demand in cholesterol for the repair of myelin and/or increased available cholesterol from degenerating myelin. Esterification may, therefore, be stimulated to avoid cytotoxic damage.

The authors acknowledge that CE represent a significantly small portion of the total cholesterol pool in the brain (globally < 0.1%)^38^, which seemingly precludes their ability to have a profound effect on cholesterol metabolism. However, a previous study in AD transgenic mice demonstrated that depletion of ACAT1 activity significantly increases levels of 24-OHC and cholesterol synthesis, demonstrating a potentially noteworthy impact on other metabolites^26^. Comparison of CE levels in this study with 24-OHC in the same human tissue^7^ reveals that control CE tissue concentrations are approximately 12% and 15% of their respective 24-OHC concentrations in the putamen and caudate. In cerebellum CE levels are equivalent to 24-OHC, which means that alterations in its abundance could affect the concentrations of other metabolites in the cholesterol pathway.

Our study also revealed specific alterations in CE between striatal subregions, with HD caudate showing a larger increase in CE than HD putamen (Fig. 2). This was unexpected since previous analysis showed no increase in cholesterol despite reduced removal via 24-OHC in HD caudate^7^. The striatum atrophies earliest of the brain regions in HD, however the caudate and putamen atrophy at different rates^39–41^, and correlate with different disease indices *i.e*. caudate with age of onset, putamen with disease severity^42^ This may contribute to the observed differences in the magnitude of lipid disturbance and in this instance, CE accumulation (Fig. 2)^2,7,18,42^. It is also possible that these regions represent different stages of cholesterol disturbance in HD, and that the caudate may have a greater capacity to maintain cholesterol balance than the putamen, possibly due to its earlier and slower degeneration in the disease^7,42^. Although a difference could not be detected between the total CE concentrations in HD and control cerebellum, the decreased abundance of several CE species is in opposition to the differences in HD striatum. Our subjects showed high variability in cerebellar lipid profiles, which has been identified in other HD studies along with variances in cerebellar degeneration leading to difficulty in statistical analysis^43^. Previous research has identified the cerebellum as an area of little cholesterol disturbance in HD, perhaps attributable to a later disturbance for this region in adult onset HD^44^. Post mortem analysis identified that reductions in cerebellar Purkinje cells were only detected in HD patients with a predominant motor phenotype, and not in mood phenotypes, indicating that the clinical expression of the patient may indicate disturbance of the cerebellum^45^. Additionally, the white matter of the cerebellum degenerates to a greater extent than the grey. Our analysis of the lipid profile of the cerebellum is consistent with that of the other grey matter regions (caudate, putamen and grey cortex) and less aligned with that of white matter regions (white cortex) indicating that the cerebellar tissue in this study was grey matter (unpublished data). We cannot therefore comment on disturbances to cholesteryl esters in the white matter of the cerebellum.

Due to the advanced stage of HD within our subjects, it is unknown if esterification of cholesterol was able to maintain homeostasis earlier on in the disease within the striatum. Previous studies have identified the early reduction of 24-OHC in HD^18,21^. This may be relevant to the maintained steady-state levels of cholesterol identified in previous HD studies despite disturbances to cholesterol metabolism. It has been hypothesized that cholesterol accumulation alters membrane organisation and cell signalling in HD striatal neurons^12,14,46^. Consideration should be made for the possible influence of medications on the brain chemistry of the subjects in this study. Some antidepressants and antipsychotics can influence the cholesterol metabolic pathway by upregulating genes that are modulated by sterol regulatory element binding proteins^47,48^. However, studies examining the effects of these drugs observed different effects on lipid metabolism dependant on the drug itself and the brain region. For example, a study looking at the effect on neural lipids after antidepressant treatment in rats found changes specific to the frontal cortex and no reported changes to lipids in the striatum or cerebellum^49^. Additionally, due to the variations in drugs prescribed among patients and the absence of clinical information regarding the duration and dosage of these drugs, analysis of their influence on our results is challenging.

This study was the first to analyse CE in human HD post-mortem tissue. Our analyses have uncovered striatal subregion specific accumulations of CE in HD. The striatal subregion specific alterations of CE in HD patients suggests an alternative mechanism of sequestration of excess cholesterol into CE, however, since no difference in the expression of ACAT1 was identified this may be a consequence of increased ACAT1 activity. This discovery highlights not only the unique disturbances of CE in the caudate and putamen but also provides further insight into the alteration of specific lipid pathways in HD pathology, which may help us to uncover the nature and specific mechanism of mhtt’s interaction with neural lipids.

## Materials and methods

### Human Post-Mortem Brain Tissue

The Victorian Brain Bank (VBB) provided human post-mortem tissue from the left hemisphere of 13 advanced stage (IV) HD subjects and 13 age and sex-matched controls. Clinical disease stage was measured using the Unified Huntington’s Disease Rating Scale. All HD cases had a pathological Vonsattel grading of IV as deemed by the Victorian Brain Bank. Tissue was selected from the caudate, putamen and cerebellum of each subject and stored at − 80 °C until use. The cerebellum was included as a comparison tissue as it is one of the least disturbed brain regions in adult-onset HD, both in terms of degeneration and its low degree of lipid disturbance^7,43^. The mean age, post mortem interval and brain pH between controls and HD subjects were not different (Table 1)^7^. Ethics approval was obtained from the UOW Human Research Ethics Committee (HE10/327) and was carried out in accordance with the Declaration of Helsinki (2008).

**Table 1:** Subject demographic information provided by the Victorian Brain Bank.

Subject	Gender	Age (years)	PMI (h)	pH
Con 1	M	78.3	46.0	6.54
Con 2	M	69.1	34.0	6.31
Con 3	M	63.9	54.5	6.51
Con 4	M	81.0	36.5	6.56
Con 5	M	64.1	24.0	6.56
Con 6	F	59.0	30.0	6.84
Con 7	F	67.3	30.0	6.23
Con 8	F	74.8	61.5	6.24
Con 9	F	68.3	71.5	6.34
Con 10	F	60.4	49.0	6.23
Con 11	M	63.9	32.0	6.47
Con 12	M	69.4	24.0	6.27
Con 13	M	75.6	46.0	6.57
Mean ± SEM		68.8 ± 1.9	41.5 ± 4.1	6.44 ± 0.05
HD 1	M	77.0	8.5	6.57
HD 2	M	68.7	72.0	6.32
HD 3	M	61.1	17.0	6.54
HD 4	M	81.1	50.5	6.23
HD 5	M	66.6	37.0	6.26
HD 6	F	57.2	22.0	6.22
HD 7	F	66.7	18.5	6.21
HD 8	F	72.2	22.0	6.43
HD 9	F	70.7	70.0	6.16
HD 10	F	51.5	63.5	6.54
HD 11	M	62.3	21.5	6.62
HD 12	M	65.9	58.0	6.33
HD 13	M	73.9	26.5	6.31
Mean ± SEM		67.3 ± 2.2	37.5 ± 6.2	6.36 ± 0.04

*PMI* post-mortem interval (*h* hours). All HD subjects determined to be Stage IV (Advanced) as measured by the Unified Huntington’s Disease Rating Scale^2,7^. *SEM* standard error of the mean.

### Lipid extraction

Lipids were extracted using a modified methyl tert-butyl ether (MTBE, HPLC grade; Fischer Scientific, Massachusetts, USA) method as described previously^50,51^. Brain tissue (10 mg) was homogenized using a bead homogenizer (FastPrep-24, MP Bio, Sydney, Australia) at 6 m/s for 40 s, using 600 mg of 1.4 mm ceramic beads in 300 µL of methanol (LC–MS grade; VWR International, Pennsylvania, USA) containing 0.01% butylated hydroxyl-toluene (BHT; Sigma Aldrich, Missouri, USA) and internal standards (2 nmol CE 22:1, Avanti Polar Lipids, Alabama, USA). The homogenate was transferred into 2 mL glass vials. MTBE (920 µL) was added to each sample and rotated for 1 h at room temperature. Ammonium acetate (Sigma Aldrich, Missouri, USA) was added (230 µL of 150 mM), samples were vortexed for 20 s before being centrifuged at 2000 × *g* for 5 min. The top organic phase was removed from each sample without disturbing the bottom aqueous phase, and transferred into a new 2 mL glass vial for storage at -20 °C. This extract was diluted 500-fold in methanol: chloroform (2:1 v/v, with 5 mM ammonium acetate) for targeted mass spectrometric analysis of CE.

### Mass spectrometry

Nanoelectrospray ionization mass spectrometry of lipid extracts was performed using a hybrid triple quadrupole linear ion trap mass spectrometer (QTRAP 5500, Sciex, Concord, Canada), equipped with an automated chip-based nanoelectrospray source (TriVersa Nanomate, Advion Biosciences, Ithaca, USA) as described previously^52^. Samples were loaded onto a 96-well plate (Eppendorf Twin-Tec 96) and sealed before direct infusion. All lipids were analysed in positive ion mode. Spray parameters were set at a gas pressure of 0.4 psi and a voltage of 1.2 kV^51,53^. Declustering potential was set to 100 V, collision cell exit potential 8 V, entrance potential 10 V and scan rate at 200 Da/s. Lipid data was acquired by targeted precursor ion scans as shown in Supplementary Table S8. Target lists for molecular lipid species within each class were generated after the manual review of spectra in Analyst (v1.6, Sciex, Ontario, Canada). Mass spectrometry data were analysed and quantified using LipidView software (v1.2, Sciex, Ontario, Canada). Processing settings were set at a mass tolerance of 0.5 Da, and minimum signal/noise of 5. Smoothing and deisotoping were enabled. Lipids were quantified by comparison of peak areas to class-specific internal standards after isotope correction^54^.

### Western blotting

Human brain samples were homogenised in buffer [0.1 M Tris–HCl, 2 mM EDTA, glycerol 10% v/v, 0.5 mM PMSF, Protease Inhibitor Cocktail (P8340, Sigma, Australia) and Phosphatase Inhibitor Cocktail 2 (Sigma, Australia)] using a bead homogeniser (Fast-Prep 24, MP Bio, Sydney, Australia) for 40 s at 6 m/s. Protein concentrations were measured using a detergent compatible protein assay (BioRad, California, USA). Samples (7.5 µg total protein) were loaded on a 4–12% Criterion Stain-Free gel (BioRad, California, USA) in triplicate, with a pooled sample run on each gel to standardise measurements between membranes. Electrophoresis was performed at 180 V for 50 min in SDS-PAGE buffer. The gels were washed for 15 min in western transfer buffer containing 20% methanol. Samples were then transferred onto 0.2 µM PVDF membranes at 100 V for 1 h. Membranes were then washed 3 × 5 min in tris-buffered saline containing Tween 20 (TBST), followed by blocking in 5% skim milk in TBST for 1 h at room temperature. Following this, membranes were washed with TBST and incubated overnight at 4 °C in the primary antibody (anti-ACAT1 [EPR10359] (ab168342), 1:20,000, Abcam, Cambridge, United Kingdom) in 1% milk in TBST. Membranes were then washed 3 × 5 min in TBST and incubated in secondary antibody (goat × anti-rabbit (AP307P) 1:5000, Merck Millipore, Massachusetts, USA) in 1% milk in TBST for 1 h at room temperature. Membranes were washed 3 × 5 min in TBST before being visualised by chemiluminescence. Membranes were then stripped for 30 min with stripping buffer (ThermoFisher, Massachusetts, USA), washed 3 × 7 min in TBST, re-blocked and incubated with housekeeper GAPDH (anti-GAPDH 1:50,000, (Rb659-060908-WS) Osenses, Keswick, Australia) before re-imaging. Expression of ACAT1 was normalised to pooled samples and GAPDH. Imaging information is provided in Supplementary Table S9.

### Statistical analysis

Outliers were identified using a 2.2 interquartile range outlier rule and excluded from further data analysis. Statistical analysis was conducted using GraphPad Prism (V5, GraphPad Software, California USA). Normality of data was assessed using a D’Agostino Pearson Omnibus test. To determine differences in CE molecular species between HD and control (Fig. 3), data fitting normality assumptions were analysed by an unpaired t-test with Welch’s correction, while data that did not fit normality assumptions were transformed or analysed by Mann–Whitney U test if the transformation was unsuccessful. A Two-Way ANOVA with a Tukey’s test was used to determine regional differences between HD and control, in total CE and ACAT1. p values less than 0.05 were considered statistically significant. Data with p < 0.01 are also indicated. Detailed statistical information is supplied in Supplementary Tables S2, S3 and S4. Data is presented as mean ± standard error of the mean (SEM). Spearman’s correlation analysis was used to examine relationships between the biometric and total CE concentrations in SPSS (v25, IBM, USA).

### Ethics approval

This research was granted ethics approval by the University of Wollongong Human Research Ethics Committee (HE10/327) and was carried out in accordance with the Declaration of Helsinki (2008).

## Supplementary information

Supplementary Information.

## Abbreviations

24-OHC: 24-Hydroxycholesterol
ACAT: Acyl coenzyme-A acyl transferase
AD: Alzheimer’s disease
ALS: Amyotrophic lateral sclerosis
CE: Cholesteryl ester
CNS: Central Nervous System
HD: Huntington’s disease
HTT: Huntingtin gene
mhtt: Mutant huntingtin
MS: Multiple sclerosis
PD: Parkinson's disease
