# Supplementary material for: Cholesteryl ester levels are elevated in the caudate and putamen of Huntington’s disease patients
Source: Sci Rep. 2020 Nov 20;10:20314. doi: 10.1038/s41598-020-76973-8 (PMC7680097; doi:10.1038/s41598-020-76973-8)
Supplement: Supplementary file 1 — Supplementary Information. [file 41598_2020_76973_MOESM1_ESM.pdf]

Supplementary Material

Cholesteryl Esters Levels are Elevated in the Caudate and Putamen of Huntington's Disease Patients

Gabrielle R. Phillips[1,2,3]; Sarah E. Hancock[4]; Simon HJ. Brown[1,3,5]; Andrew M. Jenner[6]; Fabian Kreilaus[7], Kelly Newell[1,2,3]; Todd W. Mitchell[1,2,3]

RUNNING TITLE

Cholesteryl Esters are Elevated in Huntington's Disease

AFFILIATIONS

[1]Illawarra Health and Medical Institute, Wollongong, 2522, NSW, Australia

[2]School of Medicine, University of Wollongong, Wollongong 2522, NSW, Australia

[3] Molecular Horizons, University of Wollongong, Wollongong 2522, NSW, Australia

[4] School of Medical Sciences, University of New South Wales, Sydney, 2052, NSW, Australia

[5] School of Biological Sciences, University of Wollongong, Wollongong 2522, NSW, Australia

[6] Bioanalytical Mass Spectrometry Facility, Mark Wainwright Analytical Centre, University of New South Wales, Sydney, 2052, NSW, Australia

[7]School of Medicine, Western Sydney University, Sydney, 2560, NSW, Australia

LIPID DATA AND CORRELATIONS

Supplementary Table S1 Spearman's Correlation Analyses of total CE concentrations vs Biometric Data in HD Subjects. The analysis was run using SPSS (v25). No significant correlations were found.

HD		AOD	PMI	Brain pH	Total CE (Caudate)	Total CE (Putamen)	Total CE (Cerebellum)
**AOD**	Correlation Coefficient	1.000	-0.105	-0.251	0.190	-0.080	-0.459
	Sig. (2-tailed)		0.733	0.407	0.535	0.796	0.114
	N	13	13	13	13	13	13
**PMI**	Correlation Coefficient	-0.105	1.000	-0.332	-0.204	0.107	0.149
	Sig. (2-tailed)	0.733		0.267	0.504	0.727	0.628
	N	13	13	13	13	13	13
**Brain pH**	Correlation Coefficient	-0.251	-0.332	1.000	0.020	0.104	0.124
	Sig. (2-tailed)	0.407	0.267		0.949	0.734	0.686
	N	13	13	13	13	13	13
**Total CE (Caudate)**	Correlation Coefficient	0.190	-0.204	0.020	1.000	-0.286	-0.033
	Sig. (2-tailed)	0.535	0.504	0.949		0.344	0.915
	N	13	13	13	13	13	13
**Total CE (Putamen)**	Correlation Coefficient	-0.080	0.107	0.104	-0.286	1.000	0.297
	Sig. (2-tailed)	0.796	0.727	0.734	0.344		0.325
	N	13	13	13	13	13	13
**Total CE (Cerebellum)**	Correlation Coefficient	-0.459	0.149	0.124	-0.033	0.297	1.000
	Sig. (2-tailed)	0.114	0.628	0.686	0.915	0.325	
	N	13	13	13	13	13	13

Abbreviations: **AOD** Age of Death, **CE** Cholesteryl ester, **HD** Huntington's Disease, **PMI** Post Mortem Interval.

Supplementary Table S2 Spearman's Correlation Analyses of total CE concentrations vs Biometric Data in Control Subjects. The analysis was run using SPSS (v25). No significant correlations were found.

**Control**		**AOD**	**PMI**	**Brain pH**	**Total CE (Caudate)**	**Total CE (Putamen)**	**Total CE (Cerebellum)**
**AOD**	Correlation Coefficient	1.000	0.149	0.017	0.143	-0.176	0.088
	Sig. (2-tailed)		0.627	0.956	0.642	0.566	0.775
	N	13	13	13	13	13	13
**PMI**	Correlation Coefficient	0.149	1.000	-0.310	0.105	-0.193	-0.292
	Sig. (2-tailed)	0.627		0.302	0.733	0.527	0.332
	N	13	13	13	13	13	13
**Brain pH**	Correlation Coefficient	0.017	-0.310	1.000	-0.146	0.185	0.073
	Sig. (2-tailed)	0.956	0.302		0.634	0.544	0.813
	N	13	13	13	13	13	13
**Total CE (Caudate)**	Correlation Coefficient	0.143	0.105	-0.146	1.000	0.467	0.533
	Sig. (2-tailed)	0.642	0.733	0.634		0.108	0.061
	N	13	13	13	13	13	13
**Total CE (Putamen)**	Correlation Coefficient	-0.176	-0.193	0.185	0.467	1.000	0.038
	Sig. (2-tailed)	0.566	0.527	0.544	0.108		0.901
	N	13	13	13	13	13	13
**Total CE (Cerebellum)**	Correlation Coefficient	0.088	-0.292	0.073	0.533	0.038	1.000
	Sig. (2-tailed)	0.775	0.332	0.813	0.061	0.901	
	N	13	13	13	13	13	13

Abbreviations: **AOD** Age of Death, **CE** Cholesteryl ester, **HD** Huntington's Disease, **PMI** Post Mortem Interval.

Supplementary Table S3 Spearman's Correlation Analyses of total CE concentrations vs Biometric Data. The analysis was run using SPSS (v25). No significant correlations were found.

**All Subjects**		**AOD**	**PMI**	**Brain pH**	**Total CE (Caudate)**	**Total CE (Putamen)**	**Total CE (Cerebellum)**
**AOD**	Correlation Coefficient	1.000	0.039	-0.087	0.029	-0.208	-0.252
	Sig. (2-tailed)		0.850	0.673	0.887	0.308	0.215
	N	26	26	26	26	26	26
**PMI**	Correlation Coefficient	0.039	1.000	-0.226	-0.150	-0.028	-0.017
	Sig. (2-tailed)	0.850		0.268	0.465	0.892	0.935
	N	26	26	26	26	26	26
**Brain pH**	Correlation Coefficient	-0.087	-0.226	1.000	-0.123	0.022	0.120
	Sig. (2-tailed)	0.673	0.268		0.550	0.913	0.561
	N	26	26	26	26	26	26
**Total CE (Caudate)**	Correlation Coefficient	0.029	-0.150	-0.123	1.000	0.320	0.158
	Sig. (2-tailed)	0.887	0.465	0.550		0.111	0.442
	N	26	26	26	26	26	26
**Total CE (Putamen)**	Correlation Coefficient	-0.208	-0.028	0.022	0.320	1.000	0.192
	Sig. (2-tailed)	0.308	0.892	0.913	0.111		0.348
	N	26	26	26	26	26	26
**Total CE (Cerebellum)**	Correlation Coefficient	-0.252	-0.017	0.120	0.158	0.192	1.000
	Sig. (2-tailed)	0.215	0.935	0.561	0.442	0.348	
	N	26	26	26	26	26	26

Abbreviations: **AOD** Age of Death, **CE** Cholesteryl ester, **HD** Huntington's Disease, **PMI** Post Mortem Interval.

Supplementary Table S4 Cholesteryl Esters in Caudate

	CONTROL			HD						
	**Mean**	**SEM**	**N**	**Mean**	**SEM**	**N**	**% Difference**	**Normality**	***p***	**Sig.**
**CE 15:0**	0.0041	0.0010	9	0.0051	0.0008	11	26.02	Y	0.4010	ns
**CE 16:0**	0.0166	0.0021	11	0.0293	0.0028	11	76.57	Y	0.0018	**
**CE 16:1**	0.0093	0.0014	11	0.0194	0.0027	11	108.69	Y	0.0034	**
**CE 18:0**	0.0107	0.0016	11	0.0137	0.0018	10	28.24	Y	0.2181	ns
**CE 18:1**	0.0247	0.0041	11	0.0702	0.0126	11	183.93	Y	0.0026	**
**CE 18:2**	0.0189	0.0041	11	0.0188	0.0041	11	-0.44	N	0.9487	ns
**CE 18:3**	0.0030	0.0003	10	0.0037	0.0006	11	24.42	Y	0.3165	ns
**CE 19:0**	0.0039	0.0007	11	0.0043	0.0009	11	10.91	N	0.8470	ns
**CE 19:1**	0.0017	0.0002	11	0.0021	0.0002	11	23.65	N	0.1014	ns
**CE 20:0**	0.0196	0.0025	11	0.0288	0.0054	11	47.39	Y	0.1317	ns
**CE 20:2**	0.0018	0.0004	11	0.0042	0.0008	9	133.35	N	0.0057	**
**CE 20:3**	0.0032	0.0008	11	0.0105	0.0023	10	233.35	N	0.0006	***
**CE 20:4**	0.0184	0.0025	11	0.0256	0.0031	11	39.28	Y	0.0807	ns
**CE 20:5**	0.0148	0.0018	11	0.0164	0.0011	11	10.82	N	0.3000	ns
**CE 22:4**	0.0020	0.0003	11	0.0050	0.0009	11	148.93	N	0.0024	**
**CE 22:5**	0.0050	0.0010	11	0.0078	0.0008	11	56.54	Y	0.0363	*
**CE 22:6**	0.0043	0.0007	11	0.0126	0.0021	11	194.84	Y	0.0013	**
**Total CE**	0.1607	0.0183	11	0.2745	0.0306	11	70.82	Y	0.0045	**

Data is presented in nmol/mg of brain tissue. '% Difference' refers to the percentage difference of HD compared to Controls (100*(HD MEAN-CONTROL MEAN)/CONTROL MEAN). Normality was assessed using a D'Agostino Pearson Omnibus test. All data fitting normality assumptions was tested using a two tailed unpaired t-test, whilst data not fitting assumptions was tested using a Mann Whitney U test. Abbreviations: **CE** Cholesteryl ester; **HD** Huntington's disease, **SEM** Standard Error of the Mean.

Supplementary Table S5 Cholesteryl Esters in Putamen

	CONTROL			HD						
	**Mean**	**SEM**	**N**	**Mean**	**SEM**	**N**	**% Difference**	**Normality**	***p***	**Sig.**
**CE 15:0**	0.0032	0.0007	11	0.0028	0.0003	13	-11.74	Y	0.6021	ns
**CE 16:0**	0.0156	0.0008	11	0.0203	0.0015	13	30.43	Y	0.0148	*
**CE 16:1**	0.0092	0.0011	11	0.0117	0.0009	13	26.78	Y	0.0890	ns
**CE 18:0**	0.0088	0.0008	11	0.0097	0.0009	11	10.61	Y	0.4247	ns
**CE 18:1**	0.0245	0.0024	11	0.0481	0.0062	13	96.42	Y	0.0032	**
**CE 18:2**	0.0224	0.0028	11	0.0215	0.0028	13	-3.91	Y	0.8274	ns
**CE 18:3**	0.0030	0.0003	11	0.0032	0.0003	11	6.27	Y	0.6350	ns
**CE 19:0**	0.0034	0.0006	10	0.0019	0.0001	13	-44.25	N	0.0020	**
**CE 19:1**	0.0014	0.0002	11	0.0014	0.0001	13	0.02	N	0.7762	ns
**CE 20:0**	0.0171	0.0017	11	0.0132	0.0011	13	-22.80	N	0.1056	ns
**CE 20:2**	0.0017	0.0002	7	0.0026	0.0004	11	53.90	N	0.1259	ns
**CE 20:3**	0.0036	0.0006	10	0.0075	0.0010	12	105.59	N	0.0026	**
**CE 20:4**	0.0208	0.0015	11	0.0237	0.0015	13	14.02	Y	0.1814	ns
**CE 20:5**	0.0158	0.0013	11	0.0170	0.0013	13	7.13	Y	0.5425	ns
**CE 22:4**	0.0025	0.0002	11	0.0048	0.0004	13	88.81	N	0.0001	***
**CE 22:5**	0.0035	0.0003	11	0.0051	0.0005	13	46.23	N	0.0073	**
**CE 22:6**	0.0060	0.0009	11	0.0080	0.0010	13	32.98	N	0.1191	ns
**Total CE**	0.1613	0.0086	11	0.1996	0.0118	13	23.72	Y	0.0181	*

Data is presented in nmol/mg of brain tissue. '% Difference' refers to the percentage difference of HD compared to Controls (100*(HD MEAN-CONTROL MEAN)/CONTROL MEAN). Normality was assessed using a D'Agostino Pearson Omnibus test. All data fitting normality assumptions was tested using a two tailed unpaired t-test, whilst data not fitting assumptions was tested using a Mann Whitney U test. Abbreviations: **CE** Cholesteryl ester; **HD** Huntington's disease, **SEM** Standard Error of the Mean.

Supplementary Table S6 Cholesteryl Esters in Cerebellum

	CONTROL			HD						
	**Mean**	**SEM**	**N**	**Mean**	**SEM**	**N**	**% Difference**	**Normality**	***p***	**Sig.**
**CE 15:0**	0.0044	0.0013	13	0.0020	0.0003	8	-55.15	Y	0.1732	ns
**CE 16:0**	0.0201	0.0038	13	0.0118	0.0015	9	-41.06	N	0.1264	ns
**CE 16:1**	0.0138	0.0037	13	0.0064	0.0010	9	-53.53	N	0.0364	*
**CE 18:0**	0.0130	0.0028	13	0.0070	0.0011	9	-46.01	N	0.0825	ns
**CE 18:1**	0.0259	0.0043	13	0.0182	0.0023	9	-29.90	Y	0.1745	ns
**CE 18:2**	0.0221	0.0035	13	0.0108	0.0018	9	-51.13	Y	0.0205	*
**CE 18:3**	0.0037	0.0006	13	0.0019	0.0004	9	-47.60	N	0.0111	*
**CE 19:0**	0.0034	0.0009	13	0.0017	0.0003	8	-49.08	N	0.1847	ns
**CE 19:1**	0.0023	0.0006	11	0.0017	0.0005	9	-24.99	N	0.4446	ns
**CE 20:0**	0.0209	0.0057	13	0.0155	0.0028	9	-25.45	N	0.8960	ns
**CE 20:2**	0.0022	0.0006	8	0.0017	0.0005	8	-25.21	N	0.8785	ns
**CE 20:3**	0.0023	0.0004	11	0.0020	0.0002	9	-14.50	Y	0.4683	ns
**CE 20:4**	0.0144	0.0012	13	0.0115	0.0013	9	-20.53	N	0.1102	ns
**CE 20:5**	0.0155	0.0029	13	0.0093	0.0011	9	-39.77	N	0.0825	ns
**CE 22:4**	0.0017	0.0004	11	0.0016	0.0003	9	-4.96	N	0.7664	ns
**CE 22:5**	0.0047	0.0009	13	0.0029	0.0004	9	-36.90	N	0.3933	ns
**CE 22:6**	0.0046	0.0005	13	0.0032	0.0004	9	-30.09	Y	0.0489	ns
**Total CE**	0.1730	0.0279	13	0.1087	0.0139	9	-37.18	N	0.1264	ns

Data is presented in nmol/mg of brain tissue. '% Difference' refers to the percentage difference of HD compared to Controls (100*(HD MEAN-CONTROL MEAN)/CONTROL MEAN). Normality was assessed using a D'Agostino Pearson Omnibus test. All data fitting normality assumptions was tested using a two tailed unpaired t-test, whilst data not fitting assumptions was tested using a Mann Whitney U test. Abbreviations: **CE** Cholesteryl ester; **HD** Huntington's disease **SEM** Standard Error of the Mean.

Supplementary Table S7 Spearman's correlation analyses of CE with ACAT1, Cholesterol and 24-OHC in Control and HD striatum. Analysis was conducted using GraphPad Prism (v8). No significant correlations were found. Data was provided with permission of Kreilaus et al. 2016.

		**Caudate Total CE**		**Putamen Total CE**	
		**CONTROL**	**HD**	**CONTROL**	**HD**
**ACAT1 (GADPH)**	Correlation Coefficient	0.2168	0.1319	0.0659	-0.1703
	Sig. (2 tailed)	0.4990	0.6692	0.8347	0.5786
	N	12	13	13	13
**Cholesterol**	Correlation Coefficient	-0.1469	-0.1429	-0.5495	-0.1264
	Sig. (2 tailed)	0.6509	0.6428	0.0553	0.6827
	N	12	13	13	13
**24-OHC**	Correlation Coefficient	0.1049	-0.1099	-0.3077	0.3626
	Sig. (2 tailed)	0.7493	0.7231	0.3063	0.2240
	N	12	13	13	13

Abbreviations: **24-OHC** 24-hydroxycholesterol, **ACAT1** acyl co-enzyme acyltransferase 1, **CE** Cholesteryl Ester, **HD** Huntington's Disease.

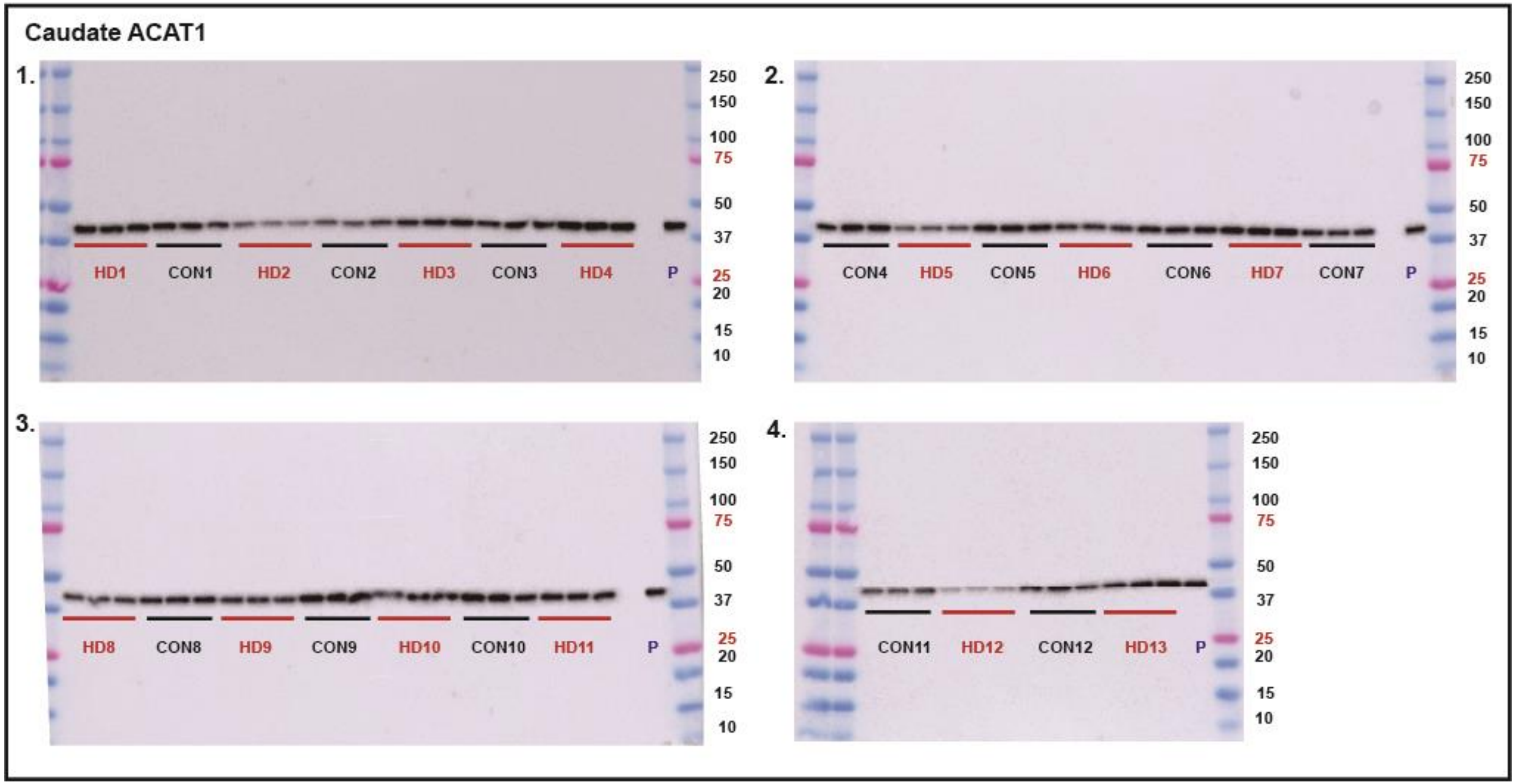

Supplementary Figure S1 ACAT 1 Detection in Caudate. Samples were loaded at 7.5 µg protein in triplicate. 10 µl of each sample was combined to make a ‘pool’ which was loaded onto each blot for standardisation. ACAT 1 ([EPR10359] (ab168342)(Recombinant)) was detected at molecular weight of ~45 kDa (Protein Marker is in kDa). Blot shown was imaged on an Amersham Gel Imager (GE LifeSciences, USA). All western blots were imaged on both the Amersham and ChemiDoc Digital Imaging systems and the best quality images are shown. All quantifications were completed using ChemiDoc images. *ACAT 1* acyl co-enzyme acyltransferase 1, *CON* Control, *HD* Huntington’s Disease, *P* Pooled Sample.

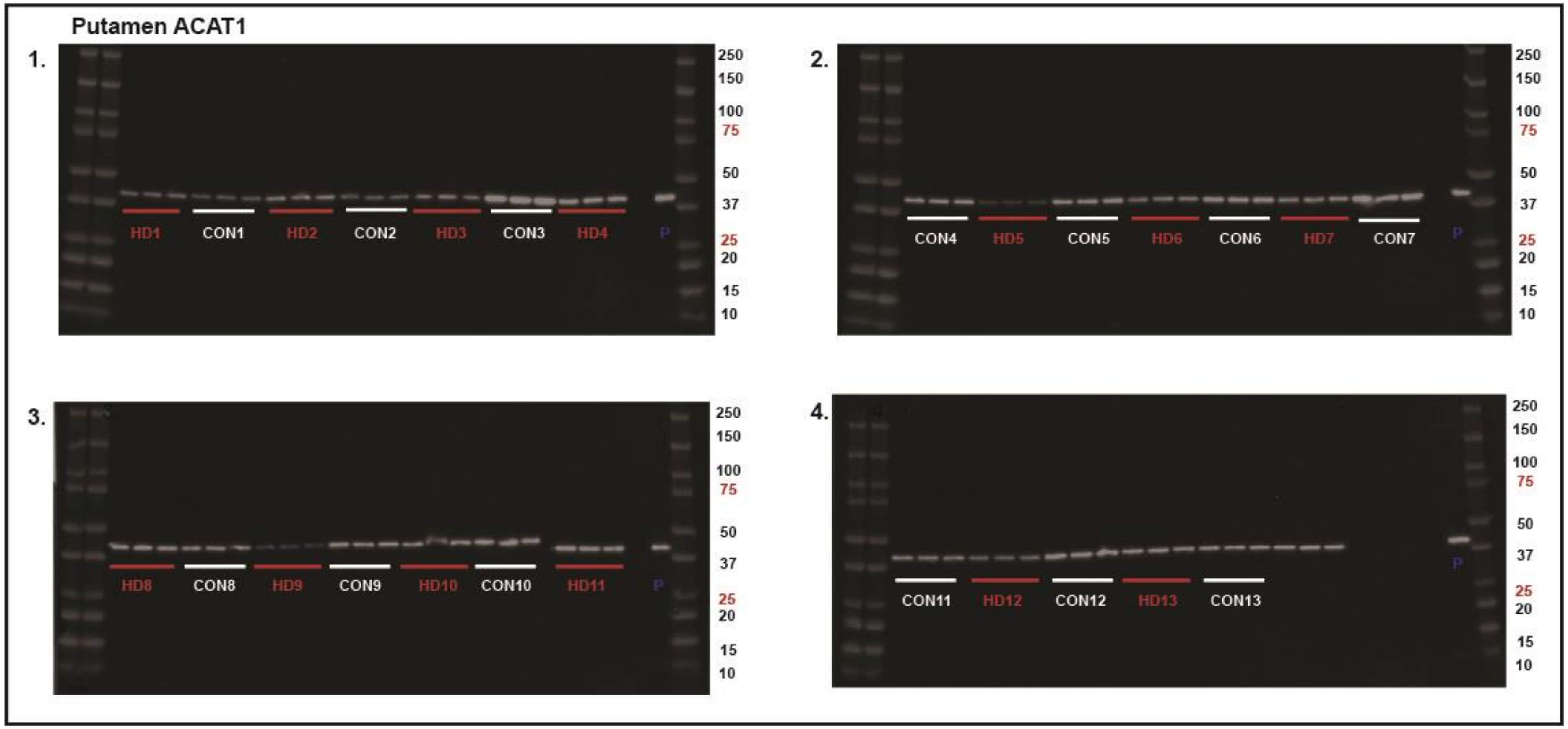

Supplementary Figure S2 ACAT 1 Detection in Putamen. Samples were loaded at 7.5 µg protein in triplicate. 10 µl of each sample was combined to make a 'pool' which was loaded onto each blot for standardisation. ACAT 1 ([EPR10359](ab168342)(Recombinant)) was detected at expected molecular weight of ~45 kDa (Protein Marker is in kDa). Blot shown was imaged on a ChemiDoc MP Digital Imager (BioRad, Massachusetts, USA). All western blots were imaged on both the Amersham and ChemiDoc Digital Imaging systems and the best quality images are shown. All quantifications were completed using the ChemiDoc images. *ACAT 1* acyl co-enzyme acyltransferase 1, *CON* Control, *HD* Huntington's Disease, *P* Pooled Sample.

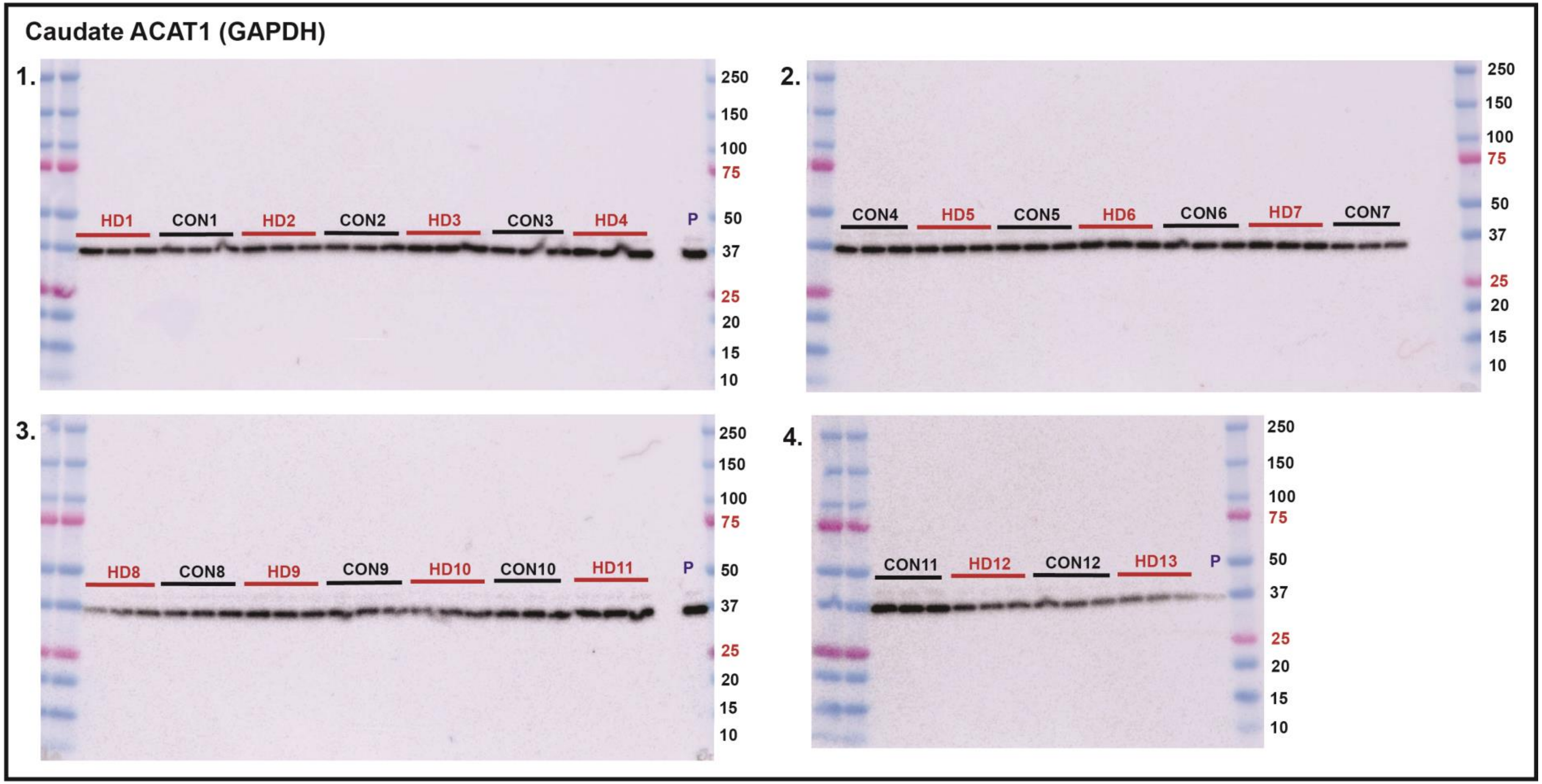

Supplementary Figure S3 GAPDH Detection for ACAT1 measurements in Caudate. Samples were loaded at 7.5 µg protein in triplicate. 10 µl of each sample was combined to make a 'pool' which was loaded onto each blot for standardisation. Blots were incubated with GAPDH (anti-GAPDH, 1:50,000, (Rb659-060908-WS), Osenses, Keswick, Australia) and used to standardise ACAT1 detection. Blot shown was imaged on an Amersham Gel Imager (GE LifeSciences, USA). All western blots were imaged on both the Amersham and ChemiDoc Digital Imaging systems and the best quality images are shown. All quantifications were completed using ChemiDoc images. *ACAT 1* acyl co-enzyme acyltransferase 1, *CON* Control, *HD* Huntington's Disease, *P* Pooled Sample.

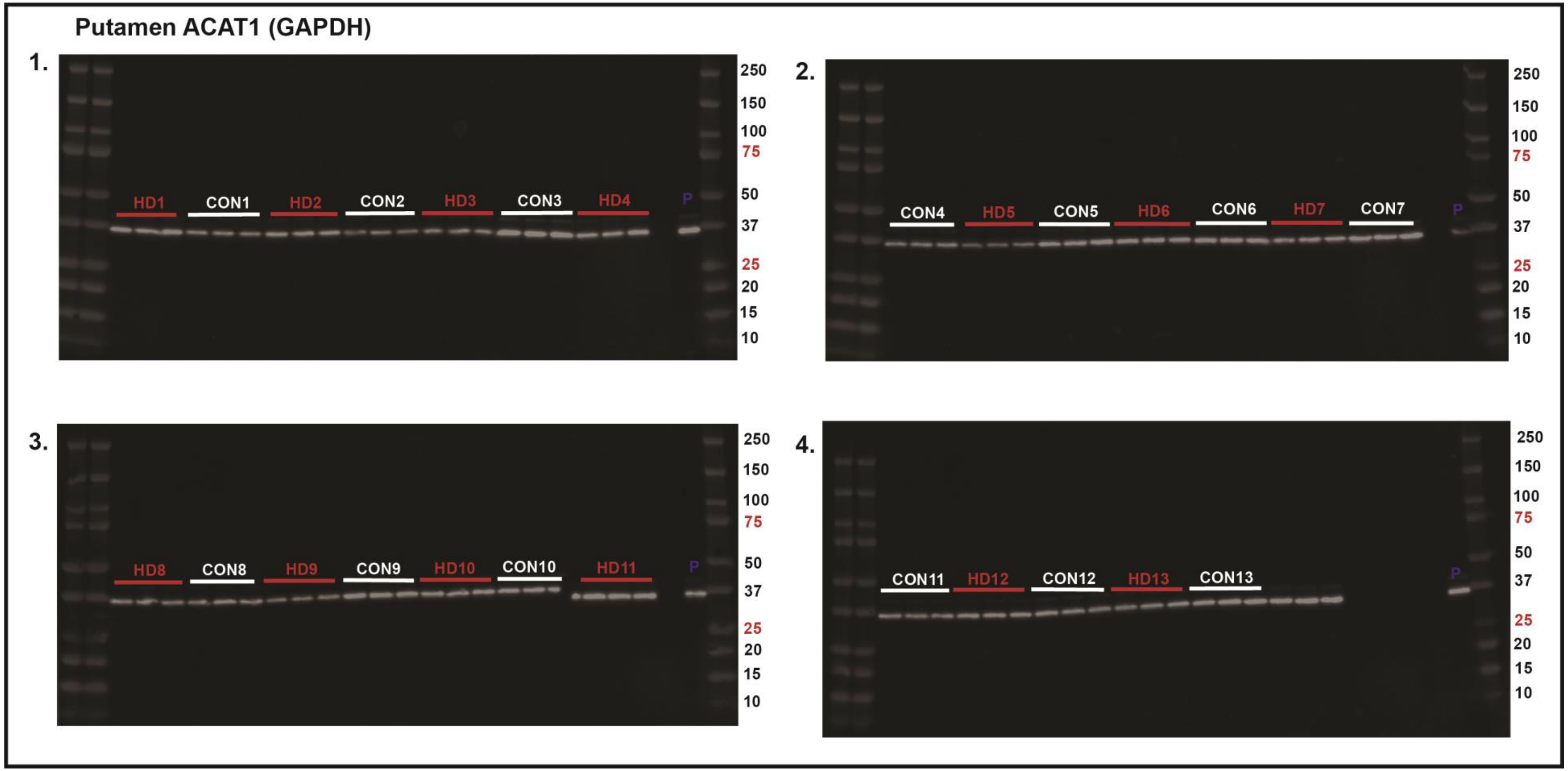

Supplementary Figure S4 GAPDH Detection for ACAT1 measurements in Putamen. Samples were loaded at 7.5 µg protein in triplicate. 10 µl of each sample was combined to make a 'pool' which was loaded onto each blot for standardisation. Blots were incubated with GAPDH (anti-GAPDH, 1:50,000, (Rb659-060908-WS), Osenses, Keswick, Australia) and used to standardise ACAT1 detection. Blot shown was imaged on an Amersham Gel Imager (GE LifeSciences, USA). All western blots were imaged on both the Amersham and ChemiDoc Digital Imaging systems and the best quality images are shown. All quantifications were completed using ChemiDoc images. *ACAT 1* acyl co-enzyme acyltransferase 1, *CON* Control, *HD* Huntington's Disease, *P* Pooled Sample.

SUPPLEMENTARY METHODS

Supplementary Table S8 Precursor scans for mass spectrometry

Lipid class	Polarity	Scan type	Collision energy (V)	Mass range (*m/z*)
**CE**	Positive	Prec *m/z* 369.4	35	590-930

Abbreviations: **CE** Cholesteryl ester

Supplementary Table S9 Antibody Information

Antibody	Product/Supplier	Species	Function	Primary	Secondary	Imaging
**anti-ACAT**	Abcam [EPR10359](ab168342)(Recombinant)	Rabbit monoclonal	Synthesis of cholesteryl esters	1:20,000 in 1% milk in TBST	1:5,000 (goat x rabbit) in 1% milk in TBST	1 minute 30 secs
**anti-GAPDH**	Osenses (Rb659-060908-WS)	Rabbit	Housekeeper (Glycolysis)	1:50,000 in 1% milk in TBST	1:5,000 (goat x rabbit) in 1% milk in TBST	1 minute
